# Volatile methyl jasmonate from roots triggers host-beneficial soil microbiome biofilms

**DOI:** 10.1038/s41589-023-01462-8

**Published:** 2023-11-13

**Authors:** Omkar S. Kulkarni, Mrinmoy Mazumder, Shruthi Kini, Eric D. Hill, Johanan Shao Bing Aow, Samantha Mun Lin Phua, Untzizu Elejalde, Staffan Kjelleberg, Sanjay Swarup

**Affiliations:** 1 Singapore Centre for Environmental Life Science Engineering (SCELSE), Singapore, Singapore; 2 Department of Biological Sciences, National University of Singapore, Singapore, Singapore; 3 Wilmar Innovation Center, Wilmar International Ltd., Singapore, Singapore; 4 NUS Synthetic Biology for Clinical and Technological Innovation (SynCTI), National University of Singapore, Singapore, Singapore; 5 School of Biological Sciences, Nanyang Technological University, Singapore, Singapore; 6 School of Biological, Earth Environmental Sciences, University of New South Wales, Sydney, New South Wales Australia; 7 Centre for Marine Science and Innovation, University of New South Wales, Sydney, New South Wales Australia; 8 NUS Environmental Research Institute, Singapore, Singapore

**Keywords:** Plant signalling, Chemical ecology, Bacteria, Plant hormones

## Abstract

The rhizosphere is a niche surrounding plant roots, where soluble and volatile molecules mediate signaling between plants and the associated microbiota. The preferred lifestyle of soil microorganisms is in the form of biofilms. However, less is known about whether root volatile organic compounds (rVOCs) can influence soil biofilms beyond the 2–10 mm rhizosphere zone influenced by root exudates. We report that rVOCs shift the microbiome composition and growth dynamics of complex soil biofilms. This signaling is evolutionarily conserved from ferns to higher plants. Methyl jasmonate (MeJA) is a bioactive signal of rVOCs that rapidly triggers both biofilm and microbiome changes. In contrast to the planktonic community, the resulting biofilm community provides ecological benefits to the host from a distance via growth enhancement. Thus, a volatile host defense signal, MeJA, is co-opted for assembling host-beneficial biofilms in the soil microbiota and extending the sphere of host influence in the rhizosphere.

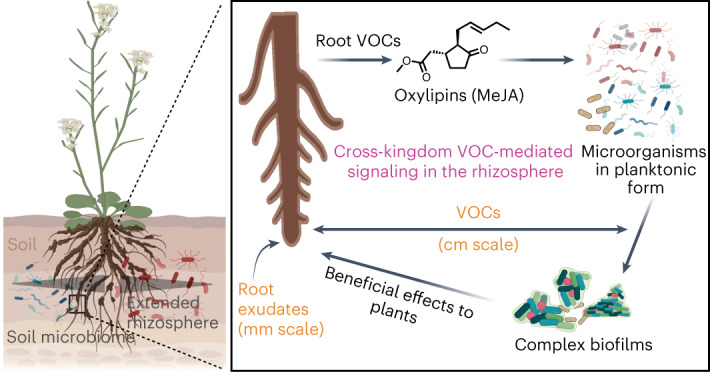

## Abstract (web summary)

Methyl jasmonate in the root volatile organic compounds (rVOCs) signals to the soil microbiome to form biofilms with altered composition that benefits plant growth. This cross-kingdom VOCs-mediated signaling expands the zone of rhizosphere influence.

## Main

A large percentage of microorganisms in nature reside in biofilms, where they are attached to a surface and embedded in a self-secreted polymeric matrix, in contrast to their free-floating or planktonic counterparts^1^. The diversity of these microorganisms is among the highest in soils, which are at least ten times higher than that of the human gut or other high-diversity environments. This high diversity is also reflected in the communities within the biofilms, which is the preferred lifestyle mode in soils for all microorganisms^2^. Multispecies biofilms in the soil can lead to emergent functions in communities that are not found in the planktonic mode for the same microorganisms^3,4^. Some such emergent functions include adhesion–cohesion capability^5^, nutrient sourcing, metabolic exchange^6,7^, communication and stability against environmental stress^8^. Plant-root-associated microorganisms are nearly 100-fold more abundant in vegetated soils than in soils lacking host influence^9^. This is mainly because plants release up to 40% of the photosynthetically fixed carbon as root exudates and volatile organic compounds (VOCs)^10^, thereby influencing the chemical ecology of root environments.

The chemical milieu around the host roots exerts a feed-forward influence in shaping the rhizosphere microbiome, which is distinct from the bulk soil microbiome^11,12^. This zone of rhizospheric influence extends 2–10 mm from the root surface^13^. Within this zone, several mediators of community assembly have been identified, such as coumarins, benzoxazinoids, salicylic acid and flavones. The resulting microbiome provides beneficial feedback to the host under both favorable and adverse environmental conditions^14^. Some of the root exudate components, such as polysaccharides^15^, fumaric acid and citric acid^16^, promote biofilm formation in individual microbial strains, mainly *Bacillus* sp. These biofilms provide beneficial functions to hosts through microorganism–microorganism interactions, leading to direct protection against pathogens^15^ and indirect protection through the induction of stress tolerance in hosts^17^. In addition, such biofilms also aid nutritional provisioning through nitrogen fixation^18^. However, compared to root exudate-mediated microbiome regulation, the impact of root VOCs (rVOCs) on the soil microbiome has been less well explored.

While root exudates are rich in soluble compounds, roots also release several rVOCs, such as terpenes, sesquiterpenes and sulfur-containing metabolites^19,20^. These VOCs have a low molecular weight (100–500 Da) and high vapor pressure^21^. These properties allow them to diffuse more readily through soil particles and have important biological roles, such as improving bacterial quorum sensing, increasing the attraction of nematodes upon attack by root-eating beetles^22^, and attracting microorganisms with antifungal properties upon fungal infection of roots^23^. However, less is known about the role of rVOCs in assembling biofilms in the soil microbiota.

In this study, we revealed bidirectional VOC-mediated biological effects between rVOCs on microbial biofilms and the effects of biofilm VOCs back on plants. We first investigated the influence of rVOCs on complex soil microbial biofilms. Using a new airflow system to direct rVOCs from plants growing in soil toward an inoculum of the soil microbiota, we discovered that rVOCs can promote biofilm formation in the soil microbiota. Next, we adopted a plant genetic approach to identify the bioactive class and the corresponding compound from rVOCs with the ability to promote biofilm formation. We confirmed the presence of the bioactive compound through targeted VOC profiling. We performed live imaging to investigate the effect of the pure bioactive compound on biofilm growth dynamics. Furthermore, several differentially abundant taxa were identified in the biofilm community in response to total rVOCs and the pure bioactive compound. Lastly, we demonstrated that the resultant biofilms could also promote plant growth from a distance.

## Results

### Plant root VOCs promote biofilms in soil microbial community

**Extended Data Fig. 1:**
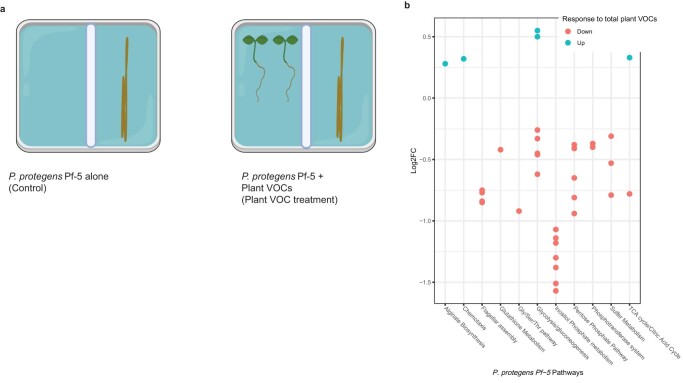
Biofilm lifestyle-related transcriptomic signature of *P. protegens* Pf-5 in response to total plant VOCs. Related to Fig. 1. **a**) Experimental design. The yellow strip in the plate represents 2 μl of 10^4^ CFU/ml of Pf-5. A 0.4 cm gap is created between these two compartments through the removal of MS agar. **b**) Statistically significant differentially abundant genes responding to total plant VOCs. P-values were calculated using negative binomial generalized linear models (adjusted p value < 0.05). Source data

To understand the effects of plant VOCs on PGPRs, we cocultured a known model PGPR, *Pseudomonas protegens* Pf-5, with *Arabidopsis* seedlings in vitro with a shared headspace without physical contact to ensure only gaseous interaction (Extended Data Fig. 1a,b). We then performed transcriptome profiling of *P. protegens* Pf-5 with and without plant VOCs 3 d postinoculation. We observed that *P. protegens* Pf-5 exposed to plant VOCs showed overall repression of metabolic pathways and flagellar motility-related genes. At the same time, we also observed a significant upregulation of certain biofilm-related genes (such as dppA—involved in chemotaxis, AlgD—involved in alginate biosynthesis) compared to that in *P. protegens* Pf-5 without plant VOCs. Such reprogramming of metabolic and motility-related genes suggests a biofilm lifestyle^24^. This led us to hypothesize that plant VOCs promote biofilm formation not only in single species but perhaps also in complex soil microbiomes.

**Fig. 1:**
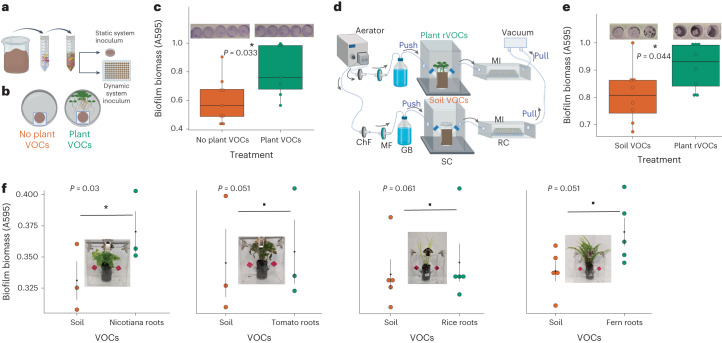
Plant root VOCs promote biofilm formation in the soil microbial community. **a**, Soil microbiota inoculum preparation. **b**, Static system for assaying the effect of plant VOCs on the microbiota. **c**, Biofilm growth quantification in the static system with volatiles from *Arabidopsis thaliana* using CV staining. Boxes range from the first (lower end) to the third quartiles (upper end), center lines denote the median values and whiskers show data lying within 1.5× interquartile range of the lower and upper quartiles. Data points at the ends of whiskers denote outliers (*n* = 7–9 independent biological replicates, *P* values calculated using two-sided parametric *t* test). **d**, Dynamic push–pull system to assay the effect of plant root volatiles (rVOCs) on the soil microbiota in the recipient chamber. **e**, Biofilm biomass quantification in the dynamic system exposed to rVOCs from *Arabidopsis thaliana* using CV staining. Boxes range from the first (lower end) to the third quartiles (upper end), center lines denote the median values and whiskers show data lying within 1.5× interquartile range of the lower and upper quartiles. Data points at the ends of whiskers denote outliers (*n* = 8 independent biological replicates, each push–pull setup is treated as one pair of biological replicates, each dot represents a biological replicate that is an average of four technical replicates and *P* values were calculated using a two-sided paired *t* test). **f**, Biofilm quantification with and without rVOCs from a variety of species (*n* = 3–4 independent biological replicates, each push–pull setup is treated as one pair of biological replicates, each dot represents a biological replicate, which is an average of four technical replicates, error bars indicate ±s.e. and *P* values were calculated using a two-sided paired *t* test); asterisk signifies *P* < 0.05 and dot signifies *P* < 0.1. Cartoons were created using the licensed version of www.biorender.com. ChF, charcoal filter; MF, microbial filter; GB, gas wash bottle; SC, source chamber; RC, receiving chamber; MI, microbiota inoculum. Source data

**Extended Data Fig. 2:**
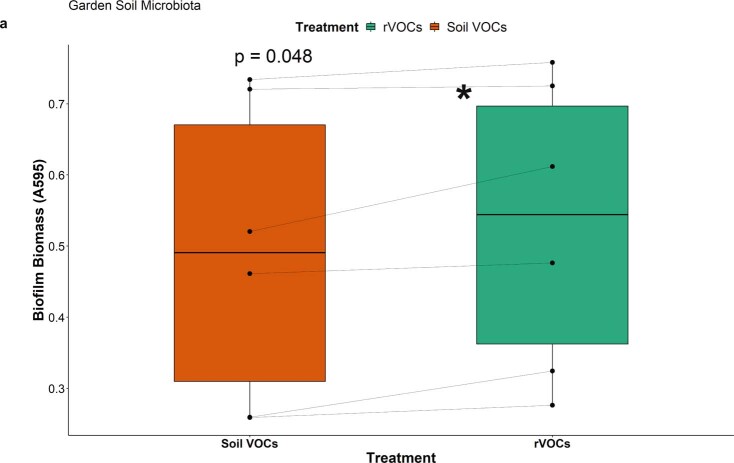
Response of garden soil microbiota to Arabidopsis rVOCs. Related to Fig. 1. **a**) Microbiota was harvested from garden soil and biofilm assay was performed in the push-pull setup and biofilm was quantified using crystal violet staining assay (same as Fig. 1d,e) (p-value calculated from paired two-sided t-tests). Source data

**Extended Data Fig. 3:**
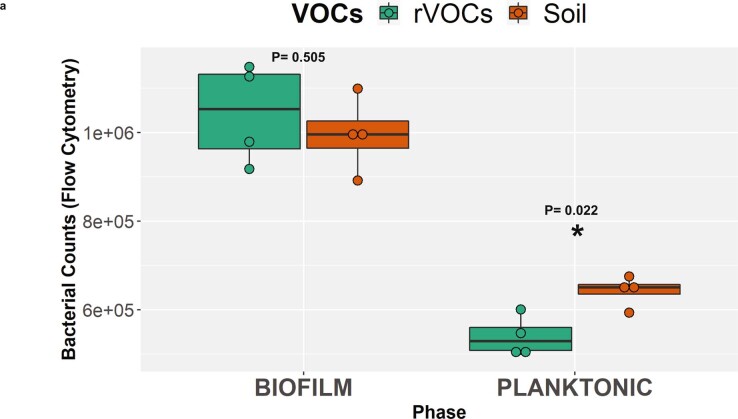
Planktonic-biofilm fractionation of soil microbiota induced by rVOCs. Related to Fig. 1. **a**) Bacterial counts as measured by flow cytometry for 2 life phases (planktonic and biofilms) of the soil microbiome inoculum exposed to plant rVOCs and soil VOCs. Boxes range from the first (lower end) to the third quartiles (upper end), center lines denote the median values and whiskers show data lying within 1.5× interquartile range of the lower and upper quartiles. Data points at the ends of whiskers denote outliers. (n = 4 independent biological replicates). Source data

To test the effect of total plant VOCs on the soil microbiome community (Fig. 1a), we first exposed the soil microbiome suspension to VOCs from 14-d-old *Arabidopsis* seedlings in a static headspace plate assay system (Fig. 1b;Static system assembly). At 24 h, the microbiota exposed to plant VOCs showed substantially higher biofilm biomass compared to the control without plants (Fig. 1c). Next, to test whether the roots were the source of these biofilm-inducing VOCs (referred to here as rVOCs), we designed a modular ‘push–pull airflow dynamic system’ (Fig. 1d and Supplementary Video 1;Dynamic system assembly) that directed sterile and humid airflow through plant roots toward the inoculum of the soil microbiome. Consistent with the phenomenon observed in the ‘static system’ assay, the microbiota exposed to rVOCs showed substantially increased biofilm biomass at 40 h (Fig. 1e). rVOC-induced biofilms were formed independently of soil types (Extended Data Fig. 2a). The lower bacterial counts in the rVOCs-exposed planktonic phase and higher bacterial counts in the rVOCs-exposed biofilm phase (Extended Data Fig. 3a) also confirmed that the observed phenomenon was not universal growth promotion of bacteria but induction of biofilm formation.

To further determine the universality of the observed phenomenon, we tested the induction of biofilms by rVOCs from different plant species spread across the plant kingdom (pteridophyte, monocots and dicots) that are separated by at least 400 million years of evolution. We used the dynamic push–pull system due to its advantage in testing VOC activity from soil-grown roots (rVOCs). In all cases, rVOCs from these diverse plant species promoted biofilm formation in the soil microbiota with slight differences in resulting trends (Fig. 1f).

### Oxylipins are the major class of rVOCs that promote biofilms

**Fig. 2:**
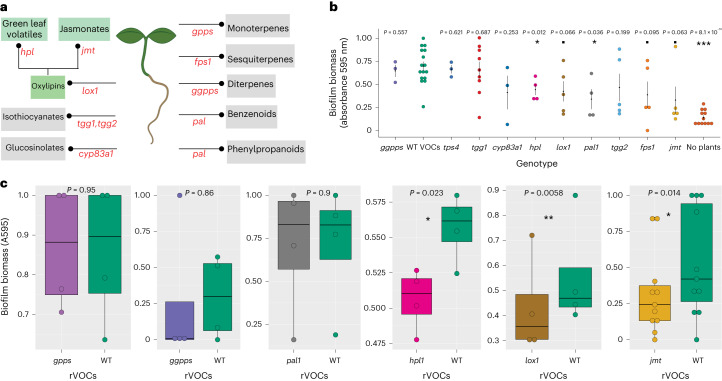
Oxylipins are the major class of root volatiles involved in biofilm promotion. **a**, Major volatile biosynthetic pathways in *Arabidopsis* and the selected biosynthetic mutant (red) gene names: *gpps*, geranyl pyrophosphate synthase; *ggpps*, geranylgeranyl pyrophosphate synthase; *tps*, terpene synthase 4; *tgg 1*/*2*, β-thioglucoside glucohydrolase-1/2; *cyp83a1*, cytochrome p450, family 83, subfamily a, polypeptide 1; *hpl*, hydroperoxide lyase; *lox1*, lipoxygenase-1; *pal1*, phenylalanine ammonialyase1; *fps1*, farnesyl pyrophosphate synthase-1; *jmt*, jasmonate methyltransferase. **b**, Crystal violet staining assay performed to screen biosynthetic mutants with biofilm promotion ability or lack thereof in the static system. The solid black dot denotes the mean, and the error bars indicate the s.e. of means (*n* = 3–16 independent biological replicates, *P* values calculated using two-sided *t* test after performing pairwise comparisons of WT rVOCs biofilms to that of individual mutants); triple asterisk signifies *P* < 0.001, single asterisk signifies *P* < 0.05 and dot signifies *P* < 0.1. **c**, Crystal violet staining assay performed on selective mutants in dynamic system. Boxes range from the first (lower end) to the third quartiles (upper end), center lines denote the median values and whiskers show data lying within 1.5× interquartile range of the lower and upper quartiles. Data points at the ends of whiskers denote outliers (each push–pull setup is treated as one pair of biological replicates, each dot represents a biological replicate that is an average of four technical replicates, error bars indicate ±s.e. and *P* values were calculated using a two-sided paired *t* test); double asterisk signifies *P* < 0.01, single asterisk signifies *P* < 0.05 and dot signifies *P* < 0.1. Source data

To identify the class(s) of plant rVOCs responsible for triggering biofilm formation in the soil microbiome, we adopted a genetic approach for screening mutant lines of *Arabidopsis* defective in the biosynthesis of the major known classes of plant VOCs, namely, benzenoids, oxylipins, phenylpropanoids, alkylglucosinolates, isothiocyanates and terpenoids (Fig. 2a). To validate the metabolite defects in the biosynthetic mutant lines, we conducted untargeted comparative profiling of rVOCs using thermal desorption–gas chromatography‒mass spectrometry (TD-GC/MS) for the WT and all the mutant lines used in our study. From the rVOCs of WT plants, 23 volatile compounds were identified after subtracting the VOCs from the soil. The rVOCs included benzenoids, fatty alcohols/oxylipins, phenylpropanoids and terpenoids, among others (Supplementary Table 1). Several rVOCs from WT plants, belonging to the fatty alcohol classes, were not detected in oxylipin biosynthetic mutants such as *lox1* and *hpl1* (Supplementary Table 1). Similarly, when compared to the WT rVOC profile, phenylpropanoids and benzenoids for *pal1* and terpenoids for *gpps* were not detected in these mutant lines, respectively. Isothiocyanates and glucosinolates were not detected among WT rVOCs. These mutants were still included in the biofilm screening as the lack of detection of these VOC classes could also be due to their low concentrations or adsorption/desorption properties.

Of the ten mutant lines defective in the biosynthesis of the previously mentioned VOC classes, only the four mutant lines defective in the oxylipin biosynthetic pathway and phenylpropanoid pathway (*lox1, hpl1, jmt* and *pal1*) produced substantially less biofilm biomass than the WT controls in the static assay system (Fig. 2b). We further tested these four mutants along with *gpps* and *ggpps* in the dynamic push–pull system for the ability of their rVOCs to induce biofilms. In this assay, only oxylipin mutants (*hpl1, jmt* and *lox1*) were unable to promote biofilm formation compared to the corresponding WT controls (Fig. 2c). Taken together, these results suggest the role of oxylipins as bioactive components of rVOCs in promoting biofilm formation in the soil microbiome.

### Methyl jasmonate is a potent rVOC that promotes biofilms

**Fig. 3:**
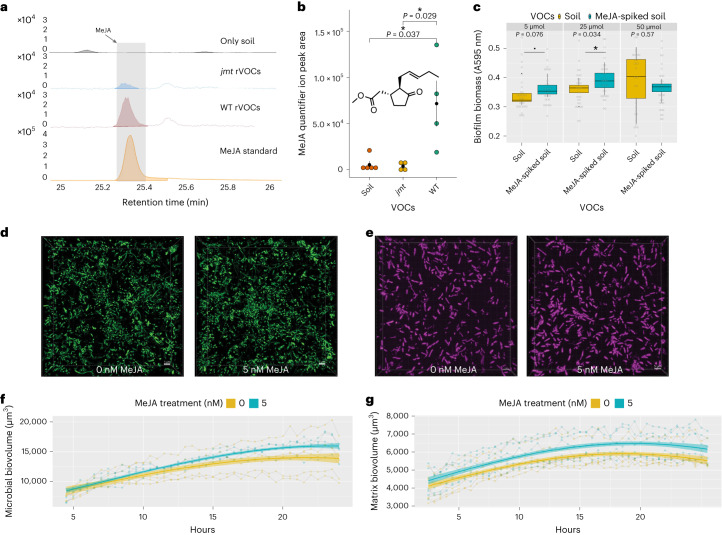
Methyl jasmonate is a potent rVOC that acts as a signal to promote biofilm formation in the soil microbiome. **a**, Extracted ion chromatogram for ions belonging to MeJA (83, 151 and 224) obtained from TD-GC/MS of VOCs from the soil, *jmt* roots and WT roots. **b**, MeJA quantifier ion (83) intensity in VOCs captured from the soil, roots of *jmt* and WT *Arabidopsis*. The solid black dot denotes the mean, and the error bars indicate the s.e. of means (*n* = 4–5 independent biological replicates; *P* values were calculated using the two-sided Wilcoxon test, asterisk signifies *P* < 0.05). **c**, Quantification of biofilms formed after treatment with different concentrations of MeJA in the push–pull dynamic system using a crystal violet staining assay. Boxes range from the first (lower end) to the third quartiles (upper end), center lines denote the median values and whiskers show data lying within 1.5× interquartile range of the lower and upper quartiles. Data points at the ends of whiskers denote outliers (boxplot and *P* values are based on four biological replicates, whereas the gray dots indicate technical replicates; *P* values were calculated by a two-sided paired *t* test using the values from biological replicates); asterisk signifies *P* < 0.05 and dot signifies *P* < 0.1. **d**, Representative snapshots of soil microbiota biofilms (nucleic acids) stained with SYTO9 treated (right) or not treated (left) with 5 nM MeJA. The scale bar denotes 5 µm (*n* = 4–5 independent biological replicates in different wells of the chamber slide). **e**, Representative snapshots of soil microbiota biofilms (extracellular matrix proteins) stained with SYPRO RUBY treated (right) or not treated (left) with 5 nM MeJA. The scale bar denotes 5 µm (*n* = 5–6 independent biological replicates in different wells of the chamber slide). **f**, Quantification of the 3D biovolume of biofilm members shown in **d**; line smoothing was performed using a linear mixed-effects model, and the faded region represents the 95% confidence interval. **g**, Quantification of the 3D biovolume of the biofilm matrix shown in **e**; line smoothing was performed using a linear mixed-effects model, and the faded region represents the 95% confidence interval. See Supplementary Tables 2 and 3 for statistical analysis. Source data

**Extended Data Fig. 4:**
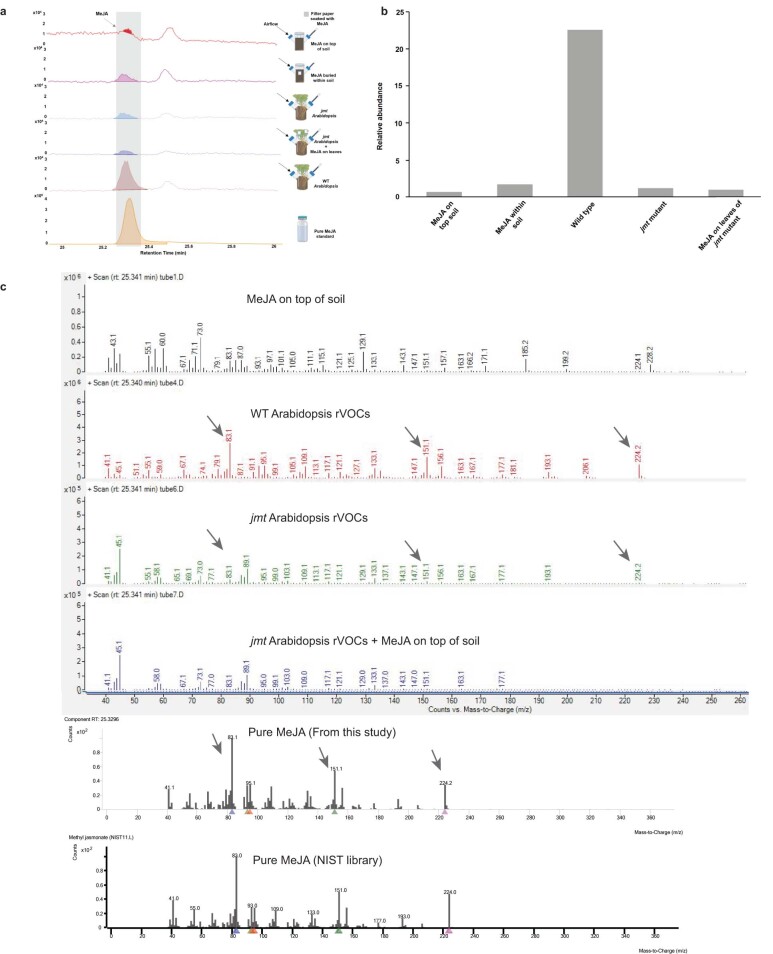
Volatile sampling system specifically samples root volatiles. Related to Fig. 3. **a**) Extracted ion chromatogram for ions belonging to methyl jasmonate (83, 151, 224) obtained from TD-GCMS of samples indicated in the left panel. White square indicates filter paper soaked with MeJA. **b**) Relative area under the curve for the peak belonging to MeJA. **c**) Mass spectra of the features detected at same retention time as MeJA detected from specific samples shown in panel a.

Methyl jasmonate (MeJA) is one of the major bioactive compounds in the oxylipin class of plant volatiles. Given the involvement of the *LOX1* and *JMT* genes in MeJA biosynthesis^25^ and the inability of their mutants to promote biofilms, we tested whether the plant roots release MeJA as a VOC. We detected the presence of MeJA in the rVOCs of *Arabidopsis* using a polymer-packed cartridge followed by direct TD (Fig. 3a and Extended Data Fig. 4a–c). MeJA levels were substantially higher in WT *Arabidopsis* seedlings than in both soil and *jmt* mutants in the dynamic system, thus showing that the MeJA detected was of plant origin (Fig. 3b). To test whether MeJA originated from roots or shoots, we soaked pieces of filter paper with MeJA and placed them above or within the soil along with *jmt* mutants (Extended Data Fig. 4a,b). The levels of MeJA detected in the rVOCs from filters placed on leaves of *jmt* mutants were comparable to those in *jmt* without the filters or in the soil alone, thereby establishing that the MeJA was released from the belowground portion of the plants.

**Extended Data Fig. 5:**
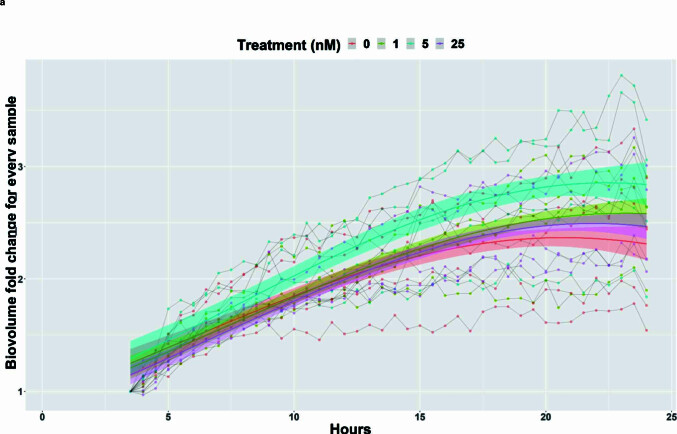
MeJA shows dose-dependent modulation of biofilm growth. Related to Fig. 3. **a**) Biofilm growth rate was calculated by normalizing the biovolume of the first frame of imaging and calculating growth compared to initial biovolume of respective samples. The line smoothing was performed using a linear mixed-effects model, and the faded region represents the 95% confidence interval. Source data

As MeJA can exist in both soluble and volatile forms, we tested its biofilm-promoting activity using both forms. To test the effect of the volatile form, we spiked 5, 25 and 50 µmol of MeJA in the soil chamber of the dynamic system setup and quantified changes in the biofilm in the recipient chamber. The potency of MeJA in biofilm promotion was gradually higher at concentrations of 5 and 25 µmol, and subsequently declined at the concentration of 50 µmol (Fig. 3c). To test the effect of the soluble form and to study the spatiotemporal dynamics of MeJA in promoting biofilms, we quantified the biovolumes of the soil microbiome and matrix of the biofilms in both time-dependent (0–24 h) and MeJA concentration-dependent (0, 1, 5 and 25 nM) manners using live confocal imaging (Fig. 3d,e, Supplementary Video 2 and Extended Data Fig. 5a). The microbiota treated with 5 nM MeJA showed increasingly higher biofilm growth compared to the control (Fig. 3f). The interaction of time and 5 nM treatment was statistically significant compared to the nontreated biofilms, as shown by the mixed-effects model (Fig. 3f and Supplementary Table 2). Similarly, 5 nM MeJA also promoted biofilm matrix formation, compared to nontreated biofilms (Fig. 3g, Supplementary Video 3 and Supplementary Table 3). The influence of MeJA appeared earlier in the matrix starting from 7 h onward, in contrast to microbial biovolumes, which differed from 15 h onward (Fig. 3f–g).

The overall results confirmed that both liquid and volatile forms of MeJA could promote biofilm formation in the soil microbiota in a dose-dependent manner, which is non-linear in nature. The results also revealed that MeJA affected both the microbial and matrix components to modulate biofilm growth dynamics.

### Biofilm community response to rVOCs is polyphyletic

**Fig. 4:**
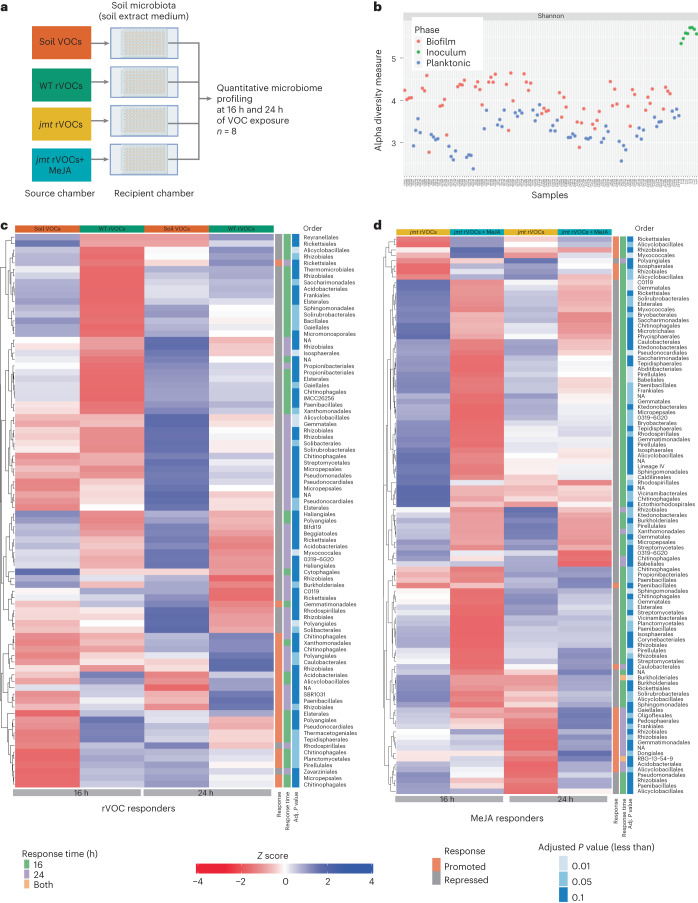
Phylogenetically diverse taxa from the soil microbiota biofilms respond to rVOCs and MeJA. **a**, Experimental design for the identification of rVOC- and MeJA responders. Boxes represent components of the push–pull airflow system. **b**, Shannon diversity of all the samples in both phases (planktonic-biofilm) and the inocula. **c**,**d**, Comprehensive heatmap of rVOC (**c**) and MeJA (**d**) responders. Source data

**Extended Data Fig. 6:**
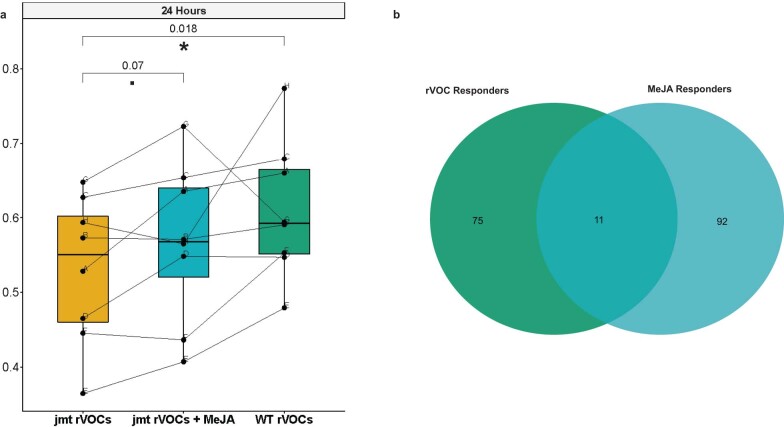
Complementation of *jmt* mutant by MeJA partially recovers ability to induce biofilms. Related to Fig. 4. **a**) Biofilm staining was performed at 16 and 24 hours. Boxes range from the first (lower end) to the third quartiles (upper end), center lines denote the median values and whiskers show data lying within 1.5× interquartile range of the lower and upper quartiles. Data points at the ends of whiskers denote outliers. (n = 8 independent biological replicates in paired groups. Grouped treatments are connected through lines) p-value calculated from paired t-test (*) signifies p<0.05, (.) signifies p<0.1 as calculated by paired *t*-test. **b**) Venn diagram showcasing the number of common and unique rVOCs and MeJA responders. Source data

**Extended Data Fig. 7:**
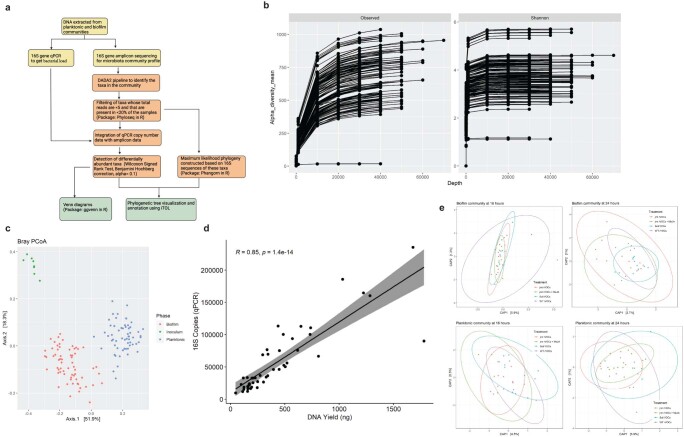
Microbiome analysis of volatile-exposed communities. Related to Fig. 4. **a**) Flowchart for data analysis used for performing quantitative microbiome profiling. **b**) Rarefaction curves showing that the read depth was sufficient to detect the existing taxa from biofilm and planktonic samples. **c**) Principal coordinate analysis (PCoA) performed by plotting Bray–Curtis distances. **d**) Pearson correlation between DNA yield and 16S copy numbers, faded region denotes 95% confidence interval. **e**, Constrained analysis of principal coordinates using Bray–Curtis distance between planktonic and biofilm communities exposed to different VOCs. The ellipsoids denote a 95% confidence interval.

Together, the following three lines of evidence, namely, (1) compromised ability to induce biofilm formation by the *jmt* mutant (Fig. 2c); (2) release of volatile MeJA from plant roots (Fig. 3a); and (3) biofilm induction by pure MeJA (Fig. 3c), indicate that root-derived volatile MeJA promotes biofilm formation in the soil microbiota. This was further corroborated by the finding that the WT rVOCs and the rVOCs from the *jmt* mutant complemented with pure MeJA (*jmt* + MeJA) were equally effective in inducing biofilm formation, suggesting that MeJA is a key factor in this process (Extended Data Fig. 6). Through 16S rRNA gene amplicon sequencing, we next investigated how taxonomically diverse members of the soil microbiome respond to WT rVOCs and MeJA (Fig. 4a). The results of the time-series analysis of biofilm growth dynamics in response to MeJA, as shown in Fig. 3f, indicated that there was an increase in biomass starting at 15 h, and this increase remained persistent at 20 h and beyond. The appearance of higher biomass at 15 h suggested that this was a critical time point in the growth process, corresponding to a stage of rapid growth or proliferation. The persistence of the higher biomass at 20 h and beyond suggested that the biofilm was able to maintain this growth rate over an extended period. Based on this information, we chose 16 and 24 h of time points as the early and late stages of biofilm formation for microbial composition analysis with sufficient sequencing depth (Extended Data Fig. 7b).

Alpha diversity was substantially higher in the biofilm than in the planktonic phases across all samples (Fig. 4b and Extended Data Fig. 7c). All biofilm communities induced due to different VOC treatments had comparable diversity (Shannon index; Supplementary Table 4). After prevalence filtering, the inoculum consisted of 1,241 amplicon sequence variants (ASVs), which differentiated into biofilm (1,039 ASVs) and planktonic (962 ASVs) lifestyles with a high degree of overlap. Across the biofilm and planktonic samples, different VOC treatments explained only 2–6% of the total variance (Extended Data Fig. 7e), implying that there was no major compositional turnover of the community within the 24-h time frame. We further investigated the changes in the abundance of specific strains in the community.

To determine the absolute abundance of each ASV in the biofilm community, we integrated the bacterial load (through qPCR) of each sample with its microbiome profile (Extended Data Fig. 7a,d). To identify the biofilm members responding to WT rVOCs, we compared the soil VOC-induced and WT rVOC-induced biofilm communities. Similarly, we identified MeJA-responsive biofilm members by comparing *jmt* rVOCs-induced and *jmt* + MeJA-induced biofilm communities.

**Extended Data Fig. 8:**
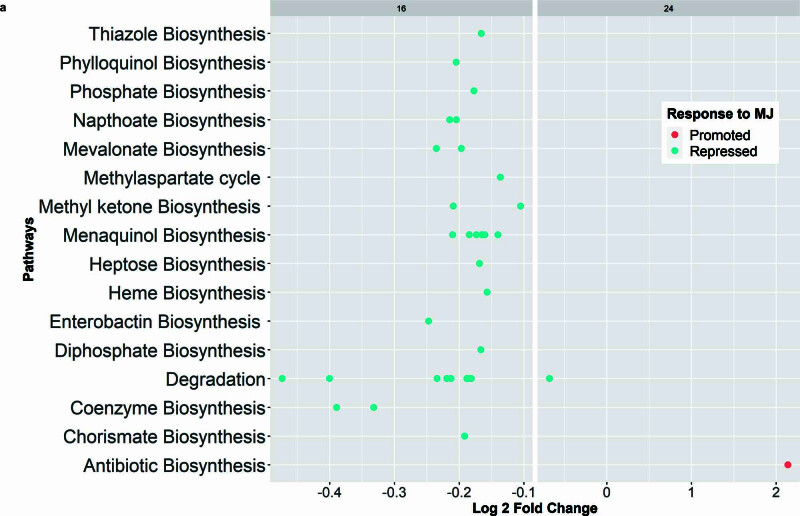
Predicted functions of biofilm community in response to MeJA exposure at 16 and 24 hours. Related to Fig. 4. **a**) Log2 fold change of gene abundances as calculated from predicted metagenomes based on 16S rRNA gene sequences. Source data

The WT rVOCs induced a significant shift in the abundance of ∼8% (86 ASVs, including 24 promoted and 62 repressed ASVs) of the biofilm community compared to those exposed to soil VOCs (Fig. 4c and Supplementary Table 5). Interestingly, the responding subcommunity differed between 16 and 24 h. MeJA also induced a significant shift in the abundance of ∼10% (103 ASVs, including 21 promoted and 82 repressed ASVs) of the biofilm community (Fig. 4d and Supplementary Table 6). Similar to rVOC responders, MeJA responders varied between 16 and 24 h, indicating rapid community succession. Apart from that, we also found that ∼15% of the total affected taxa were common between rVOCs and MeJA responders (Extended Data Fig. 6b and Supplementary Table 7). The community shift after 16 h aligns with the shift in biovolume over a similar time frame in response to MeJA (Fig. 3f). The MeJA- and rVOC-responder communities, at both 16 and 24 h, consisted mostly of repressed taxa compared to induced taxa (Fig. 4c,d). In the given context, the reduced signal for specific taxa could be due to two possible reasons such as taxa-specific DNA degradation or taxa-specific growth suppression. Determining the exact cause of the reduced signal for specific taxa would require further investigation. In response to MeJA treatment for 16 h, there was overall repression of metabolic biosynthetic pathways based on their predicted functions, which is consistent with the lifestyle shift from planktonic to biofilm mode (Extended Data Fig. 8a and Supplementary Table 8). Interestingly, this repression is lifted after the community shift occurs at 24 h (Extended Data Fig. 8a).

Overall, rVOCs and MeJA dynamically induce subtle changes in phylogenetically diverse strains within soil microbiome biofilms.

### rVOC- and MeJA-induced biofilms promote plant growth

**Fig. 5:**
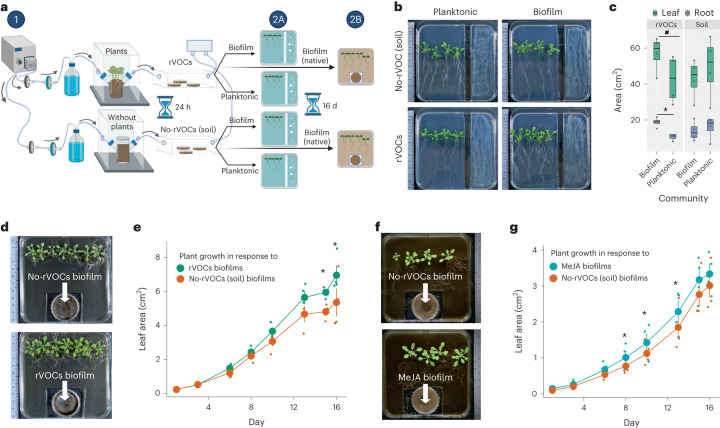
rVOC- and MeJA-induced complex biofilms promote plant growth through microbial VOCs. **a**, Experimental setup is used to study the effect of host VOC (plant rVOCs or pure MeJA)-induced biofilms on plant growth. The two-step process consists of (1) the generation of complex soil microbiome biofilms upon exposure to host VOCs over 24 h, (2A) harvesting of the microbiota from planktonic and biofilm fractions separately, followed by coculturing with 4-d-old seedlings without any physical contact and (2B) harvesting of intact biofilms (no planktonic cells) followed by co-inoculation with 4-d-old seedlings without any physical contact. In both assays, the microbiota/biofilms exposed to only soil VOCs were used as a control. Plants were continuously monitored over 16 d to obtain their digital biomass. Representative images of phenotypes resulting from exposure to the soil, root VOC-induced biofilm microbiome with shared headspace allowing gaseous exchange. **b**, Representative images of plant phenotypes resulting from coculture with non-rVOC- and rVOC-exposed planktonic and biofilm communities. **c**, Digital quantification of leaf and root area from the assay shown in **b**. Boxes range from the first (lower end) to the third quartiles (upper end), center lines denote the median values and whiskers show data lying within 1.5× interquartile range of the lower and upper quartiles. Data points at the ends of whiskers denote outliers (*n* = 4 independent biological replicates and *P* values calculated using the two-sided Wilcoxon rank sum test). **d**, Representative images of plant phenotypes resulting from coculture with intact (microbiome + matrix) biofilms without rVOCs and with rVOCs. **e**, Growth dynamics of leaf area (digital) of plants from the assay shown in **d**. The solid big dot denotes the mean, and the error bars indicate the s.e. of means (*n* = 4). *P* values were calculated using a linear mixed-effects model (random effects, each replicate; fixed effects, time and treatments). See Supplementary Table 9 for statistical analysis. **f**, Representative images of plant phenotypes resulting from exposure to intact (microbiome + matrix) biofilms without MeJA (soil) and with MeJA VOCs. **g**, Growth dynamics of leaf area (digital) of plants from the assay shown in **f**. The solid big dot denotes the mean, and the error bars indicate the standard error of means (*n* = 4). *P* values were calculated using a linear mixed-effects model (random effects: each replicate, fixed effects: time and treatments) to analyze the differences between control and host VOC biofilm-mediated plant growth. Asterisk signifies *P* < 0.05 and dot signifies *P* < 0.1. See Supplementary Table 10 for statistical analysis. Source data

**Extended Data Fig. 9:**
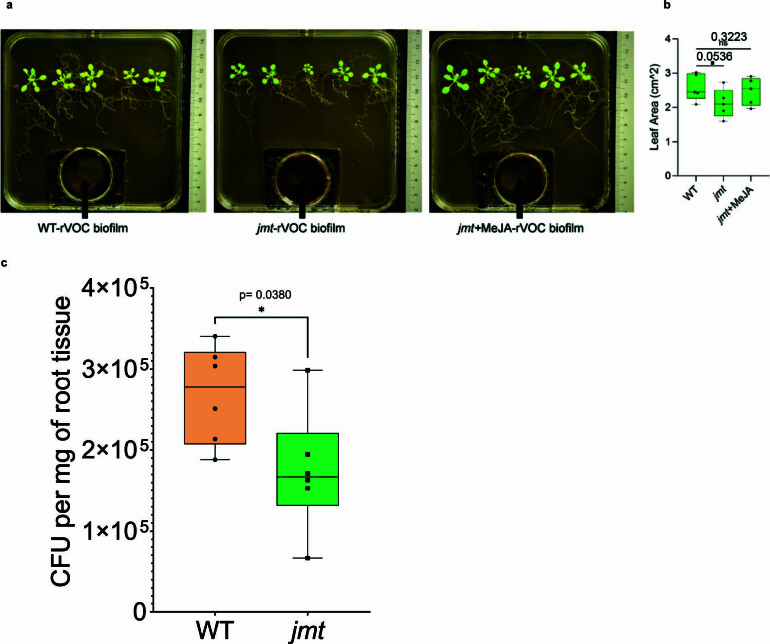
Plant growth benefits of *jmt* rVOC-induced, WT rVOCs-induced and *jmt* rVOC+ MeJA-induced biofilm and root colonization of *Bacillus* sp3. Related to Figs. 5 and 6. **a**) Photographs of Arabidopsis seedlings after 9-day exposure to complex biofilms formed in response to WT rVOCs, *jmt* rVOCs, and *jmt* rVOCs + MeJA. **b**) Quantification of leaf area of the seedlings shown in panel A. Boxes range from the first (lower end) to the third quartiles (upper end), centre lines denote the median values and whiskers show data lying within 1.5× interquartile range of the lower and upper quartiles. Data points at the ends of whiskers denote outliers. (n=5 independent biological replicates) * and ‘.’ signifies p-value less than 0.105 (two-sided Student’s t test); **c**) Positive MeJA responder strain shows compromised ability to colonize the roots of *jmt* mutant compared to WT Arabidopsis root. CFU counts of *Bacillus sp3* from Arabidopsis roots after co-culture for five days. Boxes range from the first (lower end) to the third quartiles (upper end), center lines denote the median values and whiskers show data lying within 1.5× interquartile range of the lower and upper quartiles. Data points at the ends of whiskers denote outliers. (n=6 independent biological replicates) * signifies p-value less than 0.05 (two-sided Student’s t-test). Source data

Given the evolutionarily conserved nature of the bioactivity of rVOCs on soil biofilms, we hypothesized that there would be a reciprocal ecological role of these complex biofilms on plant growth, possibly through volatile signals. To simulate the long-distance effect of MeJA-induced biofilms, we also tested their benefit to the host plants from a distance. We conducted functional in vitro assays of plant growth with the planktonic and biofilm microbiota, as well as with intact complex biofilms (Fig. 5a). After 2 weeks of coculturing, plants associated with the rVOC-induced biofilm microbiota fraction appeared to be healthy, with substantially higher root and leaf growth than the rVOC-planktonic community (Fig. 5b,c). However, soil VOC-induced planktonic and biofilm fractions did not lead to a significant difference in plant biomass. This indicated that, in the presence of plant signals, biofilm communities provide more plant growth benefits than planktonic communities. In contrast, in the absence of plant signals, biofilm and planktonic communities did not influence plant biomass yields. Hence, the plant rVOCs-influenced biofilm community showed stronger host-beneficial trait compared to the non-influenced community. Next, to study whether intact biofilms recapitulate plant growth promotion, we studied the effect of rVOC biofilms in their native form on plant growth dynamics over 2 weeks. The rVOC biofilms promoted plant growth with an increased benefit from as early as 6 d which led to significant differences from 13 d onward, compared to corresponding soil-VOC biofilms (Fig. 5d,e). Given the biofilm-promoting role of MeJA, we also tested the functionality of MeJA-induced intact biofilms on plant growth. Interestingly, these MeJA-induced biofilms also led to substantially higher leaf area than the soil VOC-induced biofilms from days 8 to 13 (Fig. 5f,g). Furthermore, we performed a plant growth assay to confirm the bioactivity of MeJA on rVOCs in inducing beneficial biofilms. We induced biofilms using rVOCs from *jmt* mutant and wild type (WT) plants and tested the plant growth benefits of these biofilms. The results showed that biofilms induced by *jmt*-rVOCs had reduced plant growth benefits compared to WT-rVOCs-induced biofilms. In the same experimental study, we also induced the biofilm using MeJA-supplemented *jmt*-rVOCs (*jmt* + MeJA) and tested the plant growth benefits of these biofilms. The results showed that MeJA could rescue the plant growth by biofilms from *jmt*-rVOCs. This finding confirmed that the absence of MeJA in rVOCs (*jmt* mutant) attenuated the beneficial property of rVOCs-induced biofilms (Extended Data Fig. 9a,b). Overall, the results of this assay provide further evidence for the role of MeJA and rVOCs in inducing beneficial biofilms that can support plant growth. As the plant growth assay lasted for 14 d in a closed system, which was a relatively long time, during which more than one factor could have contributed to the plant growth promotion. The observed plant growth benefits could have resulted from either the differences in the starting community of the biofilms (no rVOCs and rVOCs-induced biofilms) or from the shifts in the communities during this period.

### Selective MeJA responders promote plant growth from afar

**Fig. 6:**
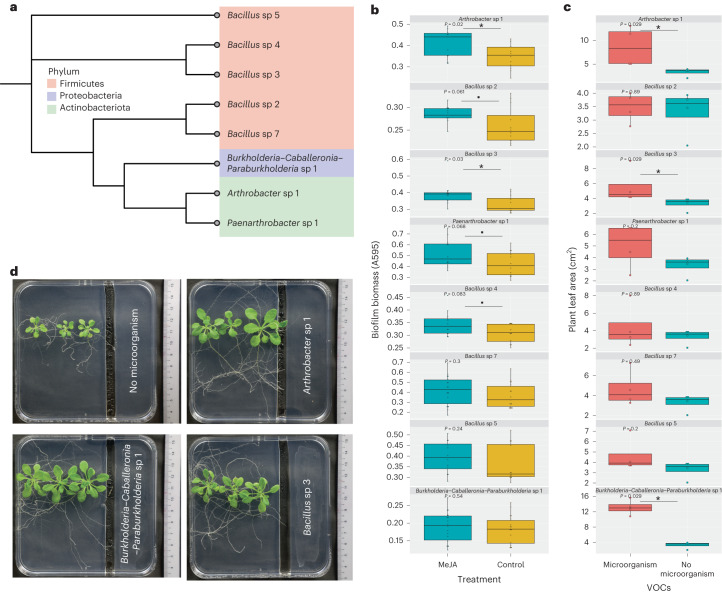
MeJA responder partially recapitulates plant growth promotion. **a**, Phylogenetic tree of microbial isolates used for this study based on their 16S sequence. **b**, Biofilm biomass of microbial isolates in response to 5 nM MeJA. Boxes range from the first (lower end) to the third quartiles (upper end), center lines denote the median values and whiskers show data lying within 1.5× interquartile range of the lower and upper quartiles. Data points at the ends of whiskers denote outliers. *P* values were calculated using a two-sided Wilcoxon rank sum test (*n* = 12–18 independent biological replicates). **c**, Effect of VOCs from microbial isolates on plant growth. *P* values were calculated using a two-sided Wilcoxon rank sum test. Boxes range from the first (lower end) to the third quartiles (upper end), center lines denote the median values and whiskers show data lying within 1.5× interquartile range of the lower and upper quartiles. Data points at the ends of whiskers denote outliers (*n* = 4 independent biological replicates). **d**, Representative pictures corresponding to the data in **c**. Source data

Plant growth promotion by complex biofilms prompted us to test whether these traits could be recapitulated at an individual strain level. We randomly selected strains cultured from MeJA-induced complex biofilms (Fig. 6a) and mapped them to the nearest ASVs in the inoculant community (ANI ≥ 97%), as described in Fig. 4. Interestingly, none of the taxa showed increments in response to MeJA or rVOCs exposure (list of ASVs), indicating the possible existence of other MeJA or rVOCs direct responders. To test this, we next determine the direct MeJA responsiveness of the remaining cultured individual strains. We tested both their MeJA responsiveness to form biofilms and plant growth promotion traits from a distance using the same assay mentioned above (Fig. 5a,b). Three of nine strains tested led to an increase in total plant leaf area by 25–300%, indicating plant growth promotion, while six were MeJA-responsive in biofilm formation (Fig. 6b,c). Interestingly, only two strains (*Arthrobacter* sp 1 and *Bacillus* sp 3) were implicated in both plant growth promotion and MeJA responsiveness. Moreover, the *Bacillus* sp 3 also showed compromised colonization in the *jmt* mutants than the WT *Arabidopsis* plants (Extended Data Fig. 9c). However, the weak correlation between MeJA responsiveness and the plant growth-promoting ability of monoculture strains, as observed in Fig. 6b,c, highlights the emergent properties of complex biofilms, where microbial community activity is more than the sum of its parts. Therefore, the same taxa in culture and community differ in their responses.

## Discussion

Here, we report a new mutually beneficial and interkingdom phenomenon in which volatiles from plant host roots induce the formation of beneficial biofilms. The presence of biofilm-inducing root volatiles across the plant kingdom, from ferns to higher plants, suggests interkingdom mutualism involving rVOCs and biofilms might be widespread. Indeed, the prevalence of bioactive root volatiles phenomenon is conserved across plants that diverged over 400 million years of evolution. What is the ecological implication of this signaling? The higher diffusability of the volatile compounds leads to a larger sphere of influence in the rhizosphere compared to a zone of 2–10 mm influenced by the soluble root exudates. Our results establish that root volatiles have a major role in shaping the rhizosphere. This study provides strong support for the growing proposals to re-evaluate the boundary of the rhizosphere^21,26^. Among the three roles of root-derived VOCs^21^, namely, hydrogen fertilization, energy metabolism and infochemicals, this study contributes to the latter by establishing the presence of an active signaling system that is effective over a long distance. The approach involving the development of a new airflow system was a key advancement leading to this discovery. The modular airflow system allows (1) flexibility in assaying the bioactivity and capturing rVOCs from diverse plant species growing in soil, (2) flexibility in assaying the effects of rVOCs on a wide range of inocula from monocultures to highly complex soil microbiomes and (3) robust experimental design to impart statistical rigor while studying chamber-based VOC interactions. As the field is nascent, researchers have recommended the laying of a strong foundation based on experimental rigor^27^, which is lacking in some recent studies.

The initial observation of biofilm promotion by rVOCs prompted us to identify the bioactive VOCs released by plant roots for this phenomenon. We adopted a genetic approach to identify the bioactive VOC classes from plant roots responsible for biofilm promotion in the soil microbiome and screened biosynthetic mutants of several VOC classes for their biofilm promotion ability. The new resource includes a comprehensive library of rVOCs from soil-grown *Arabidopsis* and its biosynthetic mutants defective in volatile pathways. Interestingly, terpenoids and phenylpropanoids, which are well known for VOC-signaling, were not involved in biofilm promotion. However, the reduced biofilm formation by several oxylipin biosynthetic mutants suggests that oxylipins are an important bioactive class of root volatiles that increase biofilm formation. This study demonstrates that host-associated oxylipins are involved in interkingdom signaling to modulate complex biofilms. Oxylipins and structurally related eicosanoids are also present in bacteria, fungi, plants and animals^28,29^. The oxylipins produced by the *Pseudomonas aeruginosa* PAO1 strain promote its own biofilm formation^30^. Within the plant kingdom, oxylipins have been reported from evolutionarily older divisions of ferns and mosses to higher plants^31^. The presence of oxylipins across the plant kingdom^31^ coupled with their involvement in biofilm promotion, as observed in our study, suggests that oxylipins are evolutionarily conserved biofilm-inducing plant signals.

This study established the following two new findings associated with an oxylipin, MeJA: its release from plant roots in volatile form and its bioactivity to promote the formation of complex biofilms in soil (Fig. 3). In this study, we observed that the biofilm induction by rVOCs seems to be evolutionarily conserved, but the bioactive signals could be either MeJA as shown for *Arabidopsis*, or other bioactive volatile molecules in other species. The role of volatile MeJA aboveground is well known in plant‒plant communication for signaling the neighboring plants to mount a defense response against herbivory and infection. Here, we established the role of MeJA belowground in plant–microorganism communication, inducing biofilm formation of soil microorganisms. Given the very low concentration of MeJA released by the roots, we posit that MeJA serves as a signaling molecule and not a nutrient molecule, which is the case for other volatile compounds. Based on the chemical properties of MeJA, it likely exists in the rhizosphere in both soluble and volatile forms. Both forms induce biofilms in the soil microbiome in a dose-dependent manner, as reported here (Fig. 3c–g), which strongly suggests a pervasive effect of this signaling both near the roots and further away from the roots, where soluble and volatile forms might be more active, respectively. While there have been elegant studies on the dynamics of root biofilm development using single strains^32,33^, this study provides a glimpse of the dynamics of a complex biofilm both with and without host influence. The altered trajectory of MeJA-induced biofilms for both microbial and matrix biovolumes suggests that microbial abundance and functions in the microbiome are likely affected. Biofilm matrix-based properties (Fig. 3f,g) that are enhanced by MeJA signaling are associated with several benefits, such as moisture retention^34^ and stability from environmental stressors^35^, which could impart ecological advantages to complex biofilms.

The altered microbial abundance and matrix production in the MeJA-induced biofilm prompted us to investigate the microbiome assembly and functions in the rhizosphere context. Within soils, host VOC (rVOCs and MeJA) signaling creates two niches of host-influenced planktonic communities and corresponding biofilms (Fig. 4), in addition to the previously reported niches of non-host-influenced soil planktonic communities and soil biofilms^36^. These host-induced biofilms have the following key characteristics: (1) the host-induced biofilm niche has a higher α diversity than the planktonic niche (Fig. 4b), which implies biofilms are the predominant reservoir of the soil microbiota. (2) Host root VOCs influence the abundance of 8–10% of the members of the biofilm communities compared to those in the non-host-influenced communities. This shift in the composition is sufficient to affect biofilm behavior, such as biovolume and matrix production, as well as the reciprocal influence on the host, as discussed below. (3) The responsiveness of the microbiome to the host signals is as rapid as 15 h, which is comparable to the dynamics of colonization directly on the root surface, although the latter studies were based on single-marked strains^32^. (4) Reduced abundances of metabolically active members at 16 h indicate a possible overall downshift of community metabolism, whereas in the second phase (24 h), this suppression is lifted (Extended Data Fig. 8). In this context, metabolic downshift is a widely reported physiological adaptation mechanism associated with biofilm formation. However, the biofilm induction by rVOCs could be due to either direct induction of biofilm pathways or indirect induction through stress pathways which, in turn, could induce biofilm formation. (5) Unlike some plant root exudates that modulate microorganisms belonging to a specific clade^37^, this phenomenon affects species belonging to diverse evolutionary clades. Overall, the affected taxa covered 19 of 24 (80%) phyla represented in host-influenced biofilms. Hence, the responders to MeJA or rVOCs appear widely distributed. This may imply that the species evolved independently to be responsive to these volatile signals. The transient and dynamic nature of shifts in the abundance of responder taxa indicates that there could either be a signaling cascade within the microbiome (early responder species influencing late responders using other molecular signals) or a universal pathway with varied response time across species (early and late responders with differential response time).

Given the marginal yet significant increase in biofilm biomass (Fig. 1c,e,f) and the rapid community succession (Fig. 4c,d) in host-induced biofilms, we investigated whether such small and transient changes could lead to larger and longer-lasting effects on host physiology. Host-induced biofilm communities have a stronger beneficial effect on a plant leaf and root growth than planktonic communities (Fig. 5b). This study demonstrates that plant signals can induce the formation of beneficial biofilms which showed a beneficial emergent property otherwise not seen in planktonic communities. Regarding the plant signals in the form of root volatiles, MeJA is a major bioactive component used to induce beneficial biofilms that can support plant growth. Moreover, MeJA-induced biofilms promoted plant growth much faster (Fig. 5f,g) than rVOC-induced biofilms (Fig. 5d,e), corroborating the ability of MeJA to enrich plant-beneficial strains in biofilms. Small changes in the plant-to-biofilm direction led to amplification in the benefits in the biofilm-to-plant direction. This is not surprising, as short-lived changes within the microbiome have been shown to have a lasting impact on the host through epigenetic regulation^38^. As a subset of MeJA-responsive biofilm isolates showed beneficial effects on plants (Fig. 6), and MeJA-triggered root colonization (Extended Data Fig. 9c), the nonbeneficial yet MeJA-responsive isolates possibly contribute in other ways to enhance the community-level functioning of the complex biofilm. This is an emergent property that arises from the interactions between different members of a complex microbial community. These properties may not be predicted or explained by studying the individual cultured strains but would require an understanding of the system as a whole.

In conclusion, a well-known plant defense signal MeJA has been co-opted to promote the formation of plant growth-promoting biofilms in the soil microbiome over an extended distance. Plants that diverged over 400 million years of evolution possess the biofilm-inducing property by root volatiles. This study contributes to the recently proposed concept^21,26^ of an ‘extended rhizosphere’ by establishing a new beneficial function in this zone. These results will have an impact on assessing and harnessing the benefits of rVOCs in regenerative agriculture through beneficial biofilms. Molecular insights into MeJA receptors and intracellular signal transduction in microorganisms will be important to gain a deeper understanding of the signaling system. In complex biofilms, the molecular basis and ecological importance of emergent properties will be highly informative in providing further insights into this new phenomenon.

## Methods

### Soil extract medium (SEM) and agar

The SEM was prepared by autoclaving 70 g of JIFFY soil substrate in 1 l of water. It was cooled and then filtered through a 0.22-µm Nalgene filtration unit. To prepare soil extract agar plates, 1% (wt/vol) agarose was added to the filtered media, and the solution was autoclaved again before pouring into the plates. This is the default broth and agar medium for all the experiments in the manuscript unless otherwise stated.

### Soil microbiota inoculum preparation

Five grams of JIFFY soil substrate was resuspended in 20 ml of PBS. This suspension was vortexed for 4 min and sonicated for 1 min. It was then filtered through a strainer. The slurry that did not pass through the strainer was again resuspended in 20 ml of PBS medium, and subsequent steps were repeated twice. The filtrate was then centrifuged at 150*g* for 2 min to settle large soil particles, and the supernatant was decanted into another tube. This supernatant was then centrifuged at 5,500*g* for 5 min to obtain a bacterial pellet. This pellet was resuspended in 20 ml of SEM. This suspension was referred to as the ‘soil microbiota inoculum’. The final concentration in all inocula was approximately 1 × 10^8^ bacteria per ml, as quantified by a Baclight bacterial counting kit (flow cytometry) or manual counting with a hemocytometer. For the confocal imaging experiments, the soil inoculum was enriched in an SEM overnight at 37 °C.

### Plant materials and growth condition

*Arabidopsis* insertional mutant lines were acquired from the Arabidopsis Biological Research Centre at Ohio State University (ABRC, http://www.arabidopsis.org/abrc/; Supplementary Table 11) and selected for homozygous lines from the population as per the instruction in ABRC wherever viable. In most cases, *Arabidopsis thaliana* (Col-0) and mutant seedlings were grown in pots with unautoclaved Jiffy Universal potting soil for up to 12 d in a plant growth chamber with the following settings: 16 h of light at 23 °C followed by 8 h of darkness at 21 °C with 80% relative humidity. Tomato, tobacco, rice and fern were also grown similarly. The plants were then transferred with their rhizosphere soil to the two-armed glass bottle in the ‘push–pull system’. To ensure an equal number of plants per pot, we first calculated the germination frequency of each mutant line, based on which we normalized the number of seeds (by weight) with WT plants Col-0. For in vitro experiments, plants were grown in soil extract agar (preparation described below). Before germination, plants were surface sterilized with 50% Chlorox and stratified for 2 d at 4 °C.

### Static system assembly

This system is a modification of the bipartite system^39^ that is routinely used to study microbial VOCs. Circular Petri plates (90 mm diameter) were filled with MS medium, and *Arabidopsis* seeds were grown on this medium (after sterilization) for 12 d. A square portion of the MS medium was cut out, and a smaller Petri plate (35 mm diameter) with microbial inoculum (1 ml) was placed in it. There was sufficient headspace to allow for gaseous exchange. The lid was then tightly closed with parafilm to avoid the loss of VOCs. At particular time points, the smaller plates were removed, and the biofilm was quantified with a crystal violet staining assay (as described below).

### Dynamic system assembly

This system is an implementation of designs proposed in the following reviews^40,41^. This system consists of an aerator/pump (to push air), a 5-µm charcoal filter (to adsorb gaseous impurities), a 0.22-µm filter (to trap microbial contamination), a gas wash bottle (to humidify the air), a source chamber (to host the source of volatiles), a recipient chamber (to receive volatiles) and a vacuum pump (to pull the air out; Fig. 1d). All the modules can be interconnected through silicone tubes to create a unidirectional continuous flow of sterile air with the help of a pump (push) and vacuum (pull). A total of 100–150 2-week-old seedlings with rhizosphere soil were kept in a customized glass pot (height, 65 mm; diameter, 45 mm) with two open side arms, which were used as the inlet and outlet for air with the roots in between. The microbiome suspension in the microtiter plate with 96 wells was kept within the recipient chamber. The whole glass pot with plants was put into the source chamber. The source chamber with only soil was used as a control to study the effects of rVOCs on soil microbiome biofilm formation. Charcoal filters (5 µm) and polytetrafluoroethylene (PTFE) filters (0.22 µm) were procured from Omega Scientific Private Limited. The source chambers and receiving chambers were custom-made by million fabricators in Singapore. Silicone tubing was used to connect all parts of the system. Airflow from the inlet (aerator) and outlet (vacuum) of the pot was measured using a mechanical flowmeter to be approximately 400 ml min^−^^1^.

### Crystal violet staining for biofilm biomass estimation

This method was repeatedly used to obtain a proxy for biofilm biomass. Briefly, planktonic cells were discarded. A volume of 50 ml of 0.1% CV solution was added to the well very gently. The biofilm was stained for 10 min. The dye was removed gently. Then, 100 µl of PBS was added to the well to wash off the excess CV. PBS was removed, and the wells were left to dry overnight. The next day, 200 µl of 1% SDS was added to each well and resuspended vigorously with a pipette. After 20 min, 20 µl of the top suspension was removed and added to a new 96-well plate. The well was diluted with 180 µl of water, and the absorbance was measured at 595 nm on a spectrophotometer.

### Volatile trapping and TD-GC/MS

Root VOCs and soil VOCs were trapped as described previously^23^. Briefly, Tenax cartridges were fitted into the one side arms of the glass pots so that their opening was exposed toward the plant roots/soil. Sterile and VOC-free air was blown from the other side of the two-armed glass pot using a push–pull system to direct the VOCs from the roots/soil to the cartridges for trapping. VOCs were sampled for 40 h and immediately analyzed by TD-GC/MS.

Sample preparation and injection were performed using the fully automated Gerstel MPS-2 autosampler and Gerstel MAESTRO software. Volatile compounds were adsorbed on a Tenax TA tube. A thermal desorption unit (TDU) was used to thermally desorb the volatiles in splitless mode at 230 °C for 10 min. To ensure that the volatiles released from the TDU were quantitatively trapped, a cooled injection system-programmed temperature vaporizer (CIS-PTV) was used. The CIS was heated from 80 °C to 230 °C at a rate of 12 °C s^−1^ with the split valve closed during sample injection into the GC inlet. Analyses of volatile compounds were performed on an Agilent 7890B GC coupled to a 5977B quadruple mass spectrometer. Separation of compounds was performed on a DB-FFAP column (60 m × 250 µm × 0.25 µm; Agilent Technologies). Helium was used as the carrier gas at a flow rate of 1.9 ml min^−1^, and solvent vent mode was used. The inlet temperature was 250 °C. The oven program was as follows: initial temperature of 50 °C held for 1 min, increased to 230 °C at the rate of 10 °C min^−1^ and held for 20 min. The temperature of the ion source and transfer line was 250 °C.

The mass spectrometer was in electron Ionization mode with an ionization energy of 70 eV, scan range of 40–300 *m*/*z* and solvent delay of 3.75 min. Analysis was performed using the MassHunter Qualified software to extract and integrate peat spectra to profile the root volatiles from WT and mutant lines. The data from only soil were considered blank and were subtracted from the rVOCs data. Compounds were identified by using the library NIST 2020 (Agilent Technologies) with a minimum hit count of 65. The compounds that were found to be present in at least two biological replicates out of three were taken into consideration for further analysis. The target compounds like MeJA were quantified by integration of peak areas and calibration using single ion monitoring (SIM) mode by monitoring the ions at 83, 151.1 and 224.1 with a dwell time of 150 ms. The peak area of these ions was considered for the relative quantification of MeJA among different samples. The raw files have been submitted to the Metabolomics Workbench^42^ server (project ID: PR001462).

### Live imaging of biofilm and matrix formation

The soil microbiota inoculum was prepared as described in the section above. MeJA was added to the microbiota to achieve the desired concentration (0, 1, 5 and 25 nM for the nucleic acid imaging experiment and 0 and 5 nM for the matrix imaging experiment). A volume of 50 ml of the microbiota suspension was added to every well of an Ibidi µ-Slide 18 Well (81816) with a cover glass bottom. A volume of 50 ml of SYTO9 (Thermo Fisher Scientific, S34854) solution (final concentration of 5 µM) was also added to all the wells. For matrix imaging, FilmTracer SYPRO Ruby Biofilm Matrix Stain (Thermo Fisher Scientific, F10318) was added instead (ready to use, 1× concentration). For live imaging, a Zeiss LSM 900 with Airyscan (Definite Focus 2) was used, and images were acquired every 30 min for 24 h with a ×65 oil objective at the NUS Centre for Bioimaging Science (CBIS). For both dyes (separate experiments), a 488-nm laser was used.

### Biofilm image analysis

Image analysis was performed using the BiofilmQ software^43^. Images were aligned along the *z* axis and over time. Two-class Otsu thresholding was used to detect the signal against the background. Sensitivity was set based on thresholding feedback. The rest of the settings were maintained as default values. Biofilm-related global properties were calculated and exported. We mainly focused on the 3D biovolume of our samples. Linear mixed effects were used to model the biovolume, where time, treatment and their interaction were the fixed effects, and every sample was considered a random effect (Tables 1 and 2). The following packages from R were used: nlme^44^, ggplot2 (ref. ^45^) and ggpubr.

### Identification of rVOC- and MeJA-responder strains

Using the push–pull airflow system, the soil microbiota inoculum was exposed to the following four VOC treatments: (1) soil VOCs; (2) WT *Arabidopsis* rVOCs; (3) *jmt* *Arabidopsis* rVOCs; and (4) *jmt* *Arabidopsis* rVOCs + MeJA. Biofilm and planktonic parts of the samples were collected from 28 wells after 16 and 24 h and stored at −80 °C. The collective sample from 28 wells was treated as a single experimental replicate. The whole experiment was repeated eight times.

### Biofilm DNA extraction and 16S rRNA gene amplicon sequencing

Biofilms were scraped at specific time points and resuspended in PBS solution. DNA‒RNA shield was added in a 1:1 ratio, and samples were stored at −80 °C. A Zymobiomics DNA miniprep kit was used to isolate DNA from the samples based on their protocol. The 16S V4-V5 region was amplified using the 515F-Y and 927R primers 45. The 20 µl reaction contained 2 µl of 10× DreamTaq buffer, 2 µl of 2 mM dNTP mix, 0.5 µl of each primer (10 µM), 0.5 µl of DreamTaq polymerase (5 U µl^−^^1^), 10 ng of template DNA and molecular grade water to make up the volume. The PCR conditions were as follows: initial denaturation at 95 °C for 3 min, 35 cycles of denaturation at 95 °C for 45 s, annealing at 50 °C for 45 s, extension at 68 °C for 90 s and final extension at 68 °C for 5 min. PCR products were purified using a Genejet PCR purification kit. The amplicon concentration was measured using a Qubit DNA BR Kit and Qubit fluorometer. 16S amplicons were submitted for next-generation sequencing on an Illumina MiSeq V3 Run (300 base pairs paired-end) at the Singapore Centre for Life Science Engineering (SCELSE). Rarefaction analysis was performed to calculate the appropriate depth for sequencing (Extended Data Fig. 7b). The sequencing raw data are deposited on the Sequence Read Archive portal (SRA: PRJNA868804).

### qPCR for 16S copies (bacterial load)

To enumerate the 16S rRNA gene copy numbers, the primers 515F^46^ and 806R^47^ were used in qPCR to amplify the 16S gene using an applied biosystem real-time PCR system. The PCR assay mixture consisted of 10 µl of PowerUp SYBR Green Master Mix, 1 µl of each primer from a 10-µM stock, 1 µl of DNA of extracted DNA from the microbial population and 7 µl of sterile nuclease-free water. The PCR amplification program encompassed an initial denaturation step at 95 °C for 3 min followed by 40 three-step cycles at 95 °C for 30 s, 52 °C for 30 s and 72 °C for 30 s. A plasmid with the fragments of the 16S rRNA gene part amplified with the same primer pair was used as the standard for creating a standard curve with a known copy number for absolute quantification. Pearson correlation was calculated for the qPCR-derived copy number and the DNA yield from all the samples (Extended Data Fig. 7d).

### Microbiome sequencing data analysis

Raw and demultiplexed sequencing data were analyzed as follows (also described in a flowchart in Extended Data Fig. 6a): primer and adapter sequences were removed using cutadapt^48^. The DADA2 (ref. ^49^) pipeline was used to learn the error rates and obtain ASVs. The Silva database was used to map the ASVs to their phylogeny. Thereafter, statistical analysis was performed as described in ref. ^50^, which included the use of Phyloseq^51^. Taxa that were present less than five times in total and present in less than 5% of the samples were removed. qPCR data were integrated with the abundance data using the script provided here^52^. The Wilcoxon signed-rank test and Benjamini–Hochberg correction were performed to compare individual ASVs in different treatments, and ASVs with an adjusted *P* value of less than 0.1 were considered statistically significant. We compared the biofilm communities exposed to *jmt* rVOCs and *jmt* rVOCs + MeJA to obtain MeJA responders (Fig. 4a,c and Supplementary Table 6). Similarly, rVOC responders (Fig. 4b and Supplementary Table 5) were identified by comparing communities exposed to soil VOCs and WT rVOCs. The phylogenetic tree was constructed using the Phangorn^53^ package and visualized using iTOL^54^. The PICRUSt 2.0 (ref. ^55^) pipeline was used to understand the predicted functions of the community. The differential functions were identified in the same way as the identification of differential taxa (integration with qPCR bacterial load data with gene tables followed by Wilcoxon rank sum test with Bonferroni–Hochberg correction). Heatmaps were plotted using the ComplexHeatmap^56^ R package.

### Effects of complex biofilms on hosts from a distance

The host benefit assay system of biofilms consists of the following two major parts (*λ*): (1) induction of biofilm with and without rVOCs/MeJA using the ‘push–pull’ system and (2) monitoring the growth of plants exposed to volatiles from induced biofilms (Fig. 5a). A volume of 2 ml of microbial inoculum was placed in a small Petri plate (35 mm) and exposed to root VOCs and soil VOCs over 24 h using a ‘push–pull dynamic’ system to generate rVOC-induced and non-rVOC-induced biofilms, respectively. After that, the planktonic fraction was gently removed to separate both the planktonic and biofilm phases of the soil inoculum of each treatment.

To assay the plant response with intact biofilms, as depicted in Fig. 5a (2B), 500 µl of fresh SEM liquid medium was added to the biofilms. A volume of 50 ml of SEM-agar (1%) was poured into square plates (120 × 120 mm) to perform the plant response assay. Part of the medium was scraped off to create space for small Petri plates with intact biofilms. The small Petri plates with biofilms were then placed with 4-d-old axenic *Arabidopsis* seedlings in shared headspace for coculturing into the growth room with the control environment. Nondestructive images were taken at regular intervals to study the growth dynamics. The leaf area was calculated using an ImageJ macro. The statistical modeling of the data was performed using linear mixed-effects models (Supplementary Tables 3 and 4).

### Isolation, biofilm assay of monoculture strains and their effect on plant growth

Complex microbiota biofilms exposed to volatile MeJA were scraped off and resuspended in PBS. Through serial dilution, this inoculum was plated on soil extract agar, minimal media agar and LB agar. Colonies with unique morphology were picked and streaked onto a fresh LB plate to acquire single colonies. The isolated strains were identified using Sanger sequencing of the PCR product with the primers 27F and 11.

Their response to soluble MeJA was tested by directly adding MeJA to the monoculture inoculum (a final concentration of 5 nM). For both assays, biofilms were stained with crystal violet and quantified after 24 h, as explained in the biofilm staining protocol. The initial OD of the inoculum was 0.2.

To test the effect of isolated strains on plant growth from a distance, a bipartite assay was performed in which 50 µl of 0.2 OD inoculum was smeared on part of the plate, and three to five seedlings (4 d old) were placed in the other part of the plate without spatial contact. Plant growth was monitored noninvasively using photography. Leaf area was quantified using an ImageJ macro as described in the previous section.

### Root colonization assay of bacterial isolate

The root colonization of the selected isolate was performed based on the method described here^57^ with some modifications. The surface-sterilized seeds of both WT and *jmt* mutant *Arabidopsis* were germinated axenically in 0.5× MS medium for 4 d. The 4-d-old seedlings were then transferred to new plates with soil extract medium (SEM) and kept in a growth room under a 16-h light/8-h dark regime at 21 °C. After 6 d, the plates were flooded with a bacterial culture resuspended (OD600-0.005) in sterile 10 mM MgCl_2_ with 0.001% Tween20. After 5 min, the individual plants were transferred to new plates with SEM and kept for another 5 d under the same growth conditions. Plants flooded with sterile 10 mM MgCl_2_ with 0.001% Tween20 without bacteria were used as controls.

To isolate and quantify the root-colonizing bacteria, roots of both WT and *jmt* plants were gently removed from the media and placed in 2 ml tubes. The roots from individual plants were weighed, rinsed and vortexed three times in 1 ml of sterile 10 mM MgCl_2_ to remove the root-associated microorganisms. The vortexed samples were then submitted to serial dilution at 1,000×. A volume of 50 ml of each dilution was plated onto LB agar plates. The colony-forming units (CFUs) were counted after 1 d of incubation at 37 °C and used to determine the original bacterial abundance per milligram of root tissue based on the root fresh weight and serial dilution used for counting.

### Reporting summary

Further information on research design is available in the Nature Portfolio Reporting Summary linked to this article.

## Online content

Any methods, additional references, Nature Portfolio reporting summaries, source data, extended data, supplementary information, acknowledgements, peer review information; details of author contributions and competing interests; and statements of data and code availability are available at 10.1038/s41589-023-01462-8.

### Supplementary information

Reporting Summary

Supplementary Tables 1–12 rVOC profile, statistical modeling for biofilm biovolume, community richness, rVOC and MeJA responders (in ASVs), common ASVs between rVOC and MeJA, predicted functions, statistical modeling of plant growth for rVOCs and MeJA, resources and *P* values.

Supplementary Videos 1–3 Dyanamic push–pull system, Syto9 live imaging and Sypro live imaging.

### Source data

Source Data Fig. 1 Statistical source data.

Source Data Fig. 2 Statistical source data.

Source Data Fig. 3 Statistical source data.

Source Data Fig. 4 Statistical source data.

Source Data Fig. 5 Statistical source data.

Source Data Fig. 6 Statistical source data.

Source Data Extended Data Fig. 1 Statistical source data.

Source Data Extended Data Fig. 2 Statistical source data.

Source Data Extended Data Fig. 3 Statistical source data.

Source Data Extended Data Fig. 5 Statistical source data.

Source Data Extended Data Fig. 6 Statistical source data.

Source Data Extended Data Fig. 8 Statistical source data.

Source Data Extended Data Fig. 9 Statistical source data.

## Data Availability

The raw sequencing data of 16S rRNA sequencing have been uploaded to Sequence Read Archive and publicly available at this link. The raw analytical data for rVOCs profiling and targeted MeJA detection have been uploaded to metabolomics workbench and publicly available, here and here, respectively. Processed data have been made available as source data in the manuscript. SILVA Database 138.1 was used in this study. Source data are provided with this paper.

## Code availability

No custom code was developed for the results in this manuscript.
