# Supplementary material for: Volatile methyl jasmonate from roots triggers host-beneficial soil microbiome biofilms
Source: Nat Chem Biol. 2023 Nov 13;20(4):473–83. doi: 10.1038/s41589-023-01462-8 (PMC10972745; doi:10.1038/s41589-023-01462-8)
Supplement: Supplementary file 1 — Reporting Summary [file 41589_2023_1462_MOESM1_ESM.pdf]

nature portfolio

Corresponding author(s): Prof Sanjay Swarup

Last updated by author(s): Sep 12, 2023

Reporting Summary

Nature Portfolio wishes to improve the reproducibility of the work that we publish. This form provides structure for consistency and transparency in reporting. For further information on Nature Portfolio policies, see our Editorial Policies and the Editorial Policy Checklist.

Statistics

For all statistical analyses, confirm that the following items are present in the figure legend, table legend, main text, or Methods section.

n/a	Confirmed	
☐	☒	The exact sample size (*n*) for each experimental group/condition, given as a discrete number and unit of measurement
☐	☒	A statement on whether measurements were taken from distinct samples or whether the same sample was measured repeatedly
☐	☒	The statistical test(s) used AND whether they are one- or two-sided<br>*Only common tests should be described solely by name; describe more complex techniques in the Methods section.*
☐	☒	A description of all covariates tested
☐	☒	A description of any assumptions or corrections, such as tests of normality and adjustment for multiple comparisons
☐	☒	A full description of the statistical parameters including central tendency (e.g. means) or other basic estimates (e.g. regression coefficient) AND variation (e.g. standard deviation) or associated estimates of uncertainty (e.g. confidence intervals)
☐	☒	For null hypothesis testing, the test statistic (e.g. *F*, *t*, *r*) with confidence intervals, effect sizes, degrees of freedom and *P* value noted<br>*Give P values as exact values whenever suitable.*
☒	☐	For Bayesian analysis, information on the choice of priors and Markov chain Monte Carlo settings
☒	☐	For hierarchical and complex designs, identification of the appropriate level for tests and full reporting of outcomes
☐	☒	Estimates of effect sizes (e.g. Cohen's *d*, Pearson's *r*), indicating how they were calculated

Our web collection on statistics for biologists contains articles on many of the points above.

Software and code

Policy information about availability of computer code

Data collection	Gas Chromatography data was collected using Agilent 7890B; Mass Spectrometry data was collected using Agilent 5977B quadruple mass spectrometer; Confocal imaging data was acquired by Zeiss LSM900; Next-Gen Sequencing data was acquired on Illumina MiSeq platform
Data analysis	Softwares used in the study are mentioned in depth in the methods section. Summary below:<br>1) For cartoon representations www.biorender.com<br>2) For statistical analysis R version 4.1.2 https://www.r-project.org/ and RStudio 2022.02.3 Build 492 (https://www.rstudio.com/)<br>3) For image analysis ImageJ 1.46r (https://imagej.nih.gov/ij/), BiofilmQ (https://drescherlab.org/data/biofilmQ/docs/)<br>4) For spectometry related data Masshunter (https://www.agilent.com/en/product/software-informatics/mass-spectrometry-software)<br>For Microbiome analysis:<br>5) DADA2 pipeline (https://benjjneb.github.io/dada2/)<br>6) Phyloseq R package 1.36.0 (https://joey711.github.io/phyloseq/)<br>7) Quantitative Microbiome Profiling (https://github.com/ raeslab/ QMP)<br>For creating phylogenetic trees<br>8) Phangorn 2.8.0 R package (https://github.com/KlausVigo/phangorn)<br>For annotating phylogenetic trees<br>9) iTOL v6 (https://itol.embl.de/)<br>For making plots<br>10) ggplot2 3.3.5 R package (https://cran.r-project.org/web/packages/ggplot2/index.html)<br>11) ggpubr 0.4.0 R package (https://cran.r-project.org/web/packages/ggpubr/index.html)<br>12) ComplexHeatmap version 2.8.0 (https://bioconductor.org/packages/release/bioc/html/ComplexHeatmap.html)<br>For statistical analysis:

13) nlme_3.1-153 R package ()https://svn.r-project.org/R-packages/trunk/nlme/

For manuscripts utilizing custom algorithms or software that are central to the research but not yet described in published literature, software must be made available to editors and reviewers. We strongly encourage code deposition in a community repository (e.g. GitHub). See the Nature Portfolio guidelines for submitting code & software for further information.

Data

Policy information about availability of data

All manuscripts must include a data availability statement. This statement should provide the following information, where applicable:

- Accession codes, unique identifiers, or web links for publicly available datasets
- A description of any restrictions on data availability
- For clinical datasets or third party data, please ensure that the statement adheres to our policy

Sequencing data:

https://www.ncbi.nlm.nih.gov/bioproject/PRJNA868804/

MeJA and rVOCs Analytical Data:

https://www.metabolomicsworkbench.org/data/DRCCMetadata.php?Mode=Study&StudyID=ST002282

Processed data has been made available as source data in the manuscript.

Databases used in this study:
SILVA Database 138.1

Human research participants

Policy information about studies involving human research participants and Sex and Gender in Research.

Reporting on sex and gender	NA
Population characteristics	NA
Recruitment	NA
Ethics oversight	NA

Note that full information on the approval of the study protocol must also be provided in the manuscript.

Field-specific reporting

Please select the one below that is the best fit for your research. If you are not sure, read the appropriate sections before making your selection.

☒ Life sciences ☐ Behavioural & social sciences ☐ Ecological, evolutionary & environmental sciences

For a reference copy of the document with all sections, see nature.com/documents/nr-reporting-summary-flat.pdf

Life sciences study design

All studies must disclose on these points even when the disclosure is negative.

Sample size	Sample size was based on the results of preliminary experiments. No statistical analysis was adopted to determine the sample size.
Data exclusions	No data exclusion
Replication	Each figure legend distinctly indicates the technical and biological replicates.
Randomization	Randomization was performed for all aspects of the experiments. For example, source/recipient chambers, arrangement of agar plates in the growth room, treatments within 96-well plate or 16-well chamber slides.
Blinding	Researchers were not fully blinded towards the treatments, partly due to the distinct phenotype of host-induced biofilms.

Reporting for specific materials, systems and methods

We require information from authors about some types of materials, experimental systems and methods used in many studies. Here, indicate whether each material, system or method listed is relevant to your study. If you are not sure if a list item applies to your research, read the appropriate section before selecting a response.

Materials & experimental systems

n/a	Involved in the study
☒	☐ Antibodies
☒	☐ Eukaryotic cell lines
☒	☐ Palaeontology and archaeology
☒	☐ Animals and other organisms
☒	☐ Clinical data
☒	☐ Dual use research of concern

Methods

n/a	Involved in the study
☒	☐ ChIP-seq
☒	☐ Flow cytometry
☒	☐ MRI-based neuroimaging
